# Relationships Between Self-Reported Sleep Quality, Quantity and Timing on Workdays vs Work-Free Days and Lifestyle Factors in Healthy Adults

**DOI:** 10.2147/NSS.S537593

**Published:** 2025-07-16

**Authors:** Diana Aslamyar, Luísa K Pilz, Charlotte von Gall

**Affiliations:** 1 Institute of Anatomy II, Medical Faculty, Heinrich Heine University, Düsseldorf, Germany; 2 Department of Anesthesiology and Intensive Care Medicine CCM | CVK and ECRC Experimental and Clinical Research Center, Charité – Universitätsmedizin Berlin, Berlin, Germany

**Keywords:** chronotype, workload, depression, anxiety, mental health, physical exercise

## Abstract

**Purpose:**

Sufficient quantity and quality of sleep are crucial for physical and mental health and performance. The ideal duration and time of sleep varies from person to person, with the latter depending on chronotype. However, rather than accommodating these needs, everyday life is often structured around rigid societal times that can result in sleep deficits and poor sleep quality. This survey study in healthy adults investigated the relationships between sleep duration, quality, and timing and how they relate to chronotype, lifestyle, perceived workload and anxiety/depression symptoms.

**Patients and Methods:**

Participants (N =315) were recruited from a large German metropolitan region. Sleep quality and quantity were evaluated separately on workdays and work-free days using assessments of tiredness upon waking and the Pittsburgh Sleep Quality Index (PSQI). Sleep time, duration, chronotype, sleep loss, and social jetlag were assessed using the Munich ChronoType Questionnaire (MCTQ). Lifestyle variables assessed in this study included exercise and substance use. Self-reported sleep quality, timing and duration were compared between work and work-free days. The relationships between variables were explored using correlation and correlation-based network analyses.

**Results:**

Our data suggest that workday sleep duration is a significant determinant of self-reported sleep quality, which in turn is negatively correlated with daytime dysfunction, anxiety/depression, and perception of workload. Moreover, physical activity and not smoking were significantly associated with self-reported sleep quality as well as with depression and anxiety symptoms.

**Conclusion:**

In addition to a healthy lifestyle, strategies to advance bedtime and/or adapt working hours to chronotype may improve sleep quality and thus mental health.

## Introduction

Adequate sleep is essential for physical and mental health, performance, and quality of life.^1–7^ Quantity and quality are fundamental components of sleep and, although the latter is difficult to define objectively.^8^ Using the Pittsburgh Sleep Quality Index (PSQI), which includes items on subjective sleep quality, sleep disruptions and continuity, daytime functioning and sleep duration, connections between insufficient sleep and depression could be shown.^9^ Sleep quantity appears to influence sleep quality, which has been shown to be more strongly related to measures of health, well-being, and sleepiness in nonclinical population.^10^ Nevertheless, both sleep quality^11^ and quantity^5^,^12^ contribute to several health, attitudinal, and affective outcomes^9^,^10^,^13^ including workload (WL) perception.^13^ In modern society, economic and social demands as well as the urge for “self-optimization” often come at the expense of sleep duration.^8^,^14^ Sleep deprivation, contributes to lifestyle-related diseases, such as metabolic and cardiovascular conditions.^7^ However, few therapeutic strategies have aimed to improve sleep duration and quality. This may be due to, among other factors, the insufficiently understood relationships and overlaps among sleep quantity, quality, and timing.

A major conceptual framework in sleep science is the “two-process model”: Sleep is regulated by a homeostatic and circadian process that interact in a complex way.^15^,^16^ Sleep timing is determined by a homeostatic sleep drive, which builds up while one is awake, and by an internally generated rhythm in sleep propensity. Like other essential body functions, sleep and wakefulness show a circadian pattern due to their regulation by the internal clock.^17^ Working hours that significantly disrupt sleep rhythms, such as night or shift work, or schedules that impose patterns such as chronic jet lag, are commonly associated with sleep deprivation. They are detrimental to mental and physical health^18^ can cause sleep disorders^19^ and are often associated with accidents^14^ and chronic derailments such as depression, cardiovascular and metabolic diseases.^2^ However, because the optimal sleep time and duration are individual, even typical school/working hours can lead to a certain irregularity in sleep-wake cycles and sleep deprivation, for example, if the morning alarm clock ends sleep prematurely. Recent studies have shown that sleep is significantly shorter and earlier on workdays than on work-free days, which may impact sleep quality.^20^,^21^

Sleep-wake times, as well as the phases (timing) of other rhythmic body functions, such as hormone secretion (including melatonin and cortisol), metabolism, and core body temperature changes, are shaped by one’s chronotype.^22^ Chronotype exists in a spectrum, ranging from extremely early-types - the colloquial “larks” - to extremely late-types – the “owls”.^23^,^24^ It changes with adolescence,^25^ has genetic determinants,^26^,^27^ and is affected by light-exposure behavior and urban lifestyles.^28^ Chronotypes probably evolved over the course of phylogeny because there is an advantage for the survival of the species if the activity is spread over a larger spectrum.^26^,^29^ However, rather than taking advantage of this wide distribution, with industrialization, societies have moved towards introducing rigid schedules. In urban areas, sleep time is also delayed, which is often associated with shorter sleep duration.^28^ With early work start times, later chronotypes tend to experience higher constraints on sleep, reflected in greater social jetlag (difference in sleep-wake timing between workdays and work-free days) and sleep deprivation on workdays. The Munich ChronoType Questionnaire (MCTQ) assesses sleep duration and sleep midpoint (MS) on workdays and workfree days,^30^,^31^ from which sleep loss (SL) and social jet lag (SJL) were derived. Chronotype, defined as an indicator of the phase of entrainment, is determined by MS on work-free days corrected for sleep debt (MSFsc).^28^ Late chronotype and social jetlag seem to be associated with pathologies^17^ and risk factors, such as consumption of alcohol, nicotine, and caffeinated drinks,^31^,^32^ as well as depression and anxiety,^33–36^ but less is known about the causal pathways.^28^ Our recent studies in a small and relatively homogeneous cohort of young healthy adults (medical students) suggest that chronotype may affect sleep quality indirectly via sleep loss^21^ and that short sleep duration on workdays is associated with higher perceived workload.^37^

Based on a previous study,^22^ our primary a priori hypothesis in this study was that self-reported sleep quality is worse on workdays than on work-free days. Furthermore, we conducted exploratory analyses to examine the associations between self-reported sleep quality and sleep duration on workdays and non-workdays, as well as chronotype, sleep loss, social jet lag with lifestyle factors, anxiety/depression, and perceived workload.

## Materials and Methods

### Procedure and Participants

The survey was conducted between November 15, 2023, and June 18, 2024. Participants were recruited through personal contacts using a snowball sampling method. Therefore, this study uses a non-probabilistic convenience sample of healthy adults who regularly work, study, or attend school, and does not use a random sample, representative of the general population. All participants were residents of the Düsseldorf metropolitan region at the time of the study, to ensure similar living conditions. Exclusion criteria for recruitment included age under 18 and over 50, no regular weekly working hours (including school and university), shift work, and a self-reported chronic illness (including sleep disorders). Upon accessing the website, participants were informed about the study conditions and data protection regulations and, after providing their consent, were redirected to the online questionnaire. This questionnaire also contained questions addressing the exclusion criteria. All questions in the online survey were mandatory and could only be answered once in one session.

The study was performed in accordance with the Declaration of Helsinki and was approved by the Research Ethics Committee of the Medical Faculty of the Heinrich Heine University (ChronoSleep study approval number: 2024–2761). All the participants provided informed consent.

### Questionnaire

The questionnaire assessed information on biological sex, age, body weight and height, general health and work habits, lifestyle, subjective workload, health, sleeping habits, and self-reported sleep quality during the last four weeks. The body mass index was calculated by dividing the body weight in kilograms by the square of the height in meters. The set of questions about lifestyle included items on physical activity, including walking and cycling for at least 20 minutes, and on the consumption of drugs, cigarettes/e-cigarettes, alcohol, and caffeine. Items on the consumption of drugs and cigarettes/e-cigarettes were scored on a two-point scale (no=0, yes=1). The items on physical activity and consumption of alcohol and caffeinated drinks were scored on a five-point scale (none =0, 1–2 times per week=1, several times a week=2, once a day=3, and several times a day=4). The workload question asked how high the participants generally rated their workload on a four-point scale (low=0, moderate=1, high=2, and very high=3).

We also used the 4-item patient health Questionnaire for Depression and Anxiety Symptoms (PHQ-4; Lowe et al (2010)).^38^ Each item of the PHQ-4 was scored on a four-point scale (not at all=0, several days=1, more than half the days=2, nearly every day=3). The total score was determined by summing the scores for the four items. Higher scores indicate higher levels of anxiety and depressive symptoms and are rated as normal (0–2), mild (3–5), moderate (6–8), or severe (9–12).^38^

Participants’ sleeping habits and self-reported sleep quality were assessed separately for workdays (w) and work-free (f) days^21^ and the questionnaires referred to the past four weeks. Questions included when they went to bed and when they awoke, how long it took them to fall asleep, and if they used an alarm clock, according to the core MCTQ^24^ (Copyright permission has been granted by Prof. Till Roenneberg). Sleep duration (SD), midpoint of sleep (MS), and chronotype were calculated based on self-reported sleep and wake-up times. Sleep loss (SL) was calculated by subtracting the sleep duration on workdays from that on work-free days as described earlier.^21^,^39^ Social jetlag (SJL) was calculated by subtracting the midpoint of sleep on workdays from the midpoint of sleep on work-free days.^24^ Chronotype, expressed in local time, was calculated based on the midpoint of sleep on work-free days, corrected for oversleeping if individuals sleep longer on work-free days than on workdays (MSFsc).^39^,^40^

Self-reported sleep quality was assessed with a question on how tired participants felt when waking in the morning on a three-point scale (rested=0, tired=1, very tired=2)^21^,^39^ and with the Pittsburgh Sleep Quality Index (PSQI).^9^ These were asked separately for workdays and work-free days.^21^ Higher scores indicate worse sleep quality. The PSQI consists of ten questions answered on a 4-point scale. Seven components were derived from the responses, each scored on a 4-point scale, and summed to produce a global score ranging from 0 to 21. Some of the response options (less than once a week, once or twice a week, three or more times a week) in the original version^9^ and in an earlier version^21^ are not optimal, considering the different proportions of work- and work-free days within a week. Therefore, we adapted them to match the response options of the PHQ-4 questionnaire (not at all=0, several days=1, more than half of the days=2, nearly every day=3).

### Sample Size Calculation

Based on previous results^21^ of PSQI comparisons between work- and work-free days, we assumed an effect size of 0.18. With a statistical power of 1-ß=0.95, the sample size of n=316 was calculated for a nonparametric Wilcoxon signed-rank test. Since we expected an exclusion rate of 5%, 369 subjects were recruited.

### Data Analysis

Statistical analysis was performed using Prism Version 7.01 (GraphPad), R (R Core Team) with R studio (Version 2023.12.0+369) and the R-packages *corrr*^41^ and *corrplot*.^42^ Data normality was tested using the D’Agostino and Pearson normality test. Because not all variables followed a normal distribution, non-parametric tests were used. Accordingly, the data were expressed as medians with interquartile range (Q1-Q3). We used the Wilcoxon matched-pairs signed-rank test to compare the variables between workdays and work-free days. Statistical significance was set at P< 0.05. To investigate the strength and direction of the relationships between ordinal variables, Spearman’s rank correlations were computed with a 95% confidence interval. The Holm-Bonferroni correction was used to reduce the probability of Type I errors from multiple correlations within each correlation matrix presented. For nominal variables, such as sex and smoking, associations were examined using Mann–Whitney *U*-test followed by the Bonferroni correction for multiple comparisons within each group comparison (eg, PSQI score by smoking status – y/n, where 2 tests were conducted: one comparing PSQI_w and one comparing PSQI_f, m = 2). Network analysis was performed based on the significant Spearman correlations using the network plot function in the *corr* R package (min_cor = 0.11).

## Results

### Sample Size

Of the 369 subjects recruited, we had to exclude 9 for meeting least one exclusion criterion based on the questionnaire (two being older than 50 years, three being shift-workers, four having a chronic disease). In addition, nine participants had to be excluded as they did not answer all questions. This resulted in a final sample size of 351.

### General Characteristics

The distributions of age, sex, and BMI, according the WHO classification,^43^ are shown in Table 1. The distribution of physical activity frequency and consumption of alcoholic and caffeinated beverages, cigarettes/e-cigarettes, and drugs are shown in Table 2. Correlations among general characteristics and life style factors see Supplementary Figure 1a, correlations between life style factors and MCTQ-derived variables see Supplementary Figure 1b, correlations between general characteristics and sleep variables see Supplementary Figure 1c. The distributions of subjective workload and PHQ-4 scores are presented in Table 3.

**Table 1:** Sex, Age and BMI (kg/m^2^) Distribution (n=351)

	n	% of Total
**Sex**		
Female	191	54
Male	160	46
**Age (years)**		
18-27	241	68
28-37	83	23
38-47	27	8
**BMI Category (kg/m2)**		
Underweight (<18.5)	23	7
Normal weight (18.5–24.9)	214	61
Pre-obesity (25.0–29.9)	80	23
Obesity class I (30.0–34.9)	28	8
Obesity class II (35.0–39.9)	4	1
Obesity class III (≥ 40.0)	2	1

**Table 2:** Distribution of the Scores for Physical Activity Frequency and Consumption of Alcoholic and Caffeinated Beverages, Cigarettes/e-Cigarettes, and Drugs (n=351)

	n	% of Total
**Physical activity**		
No	96	27
1-2 per week	107	30
Several times per week	109	31
Once a day	25	7
Several times a day	14	4
**Consumption of alcohol**		
No	228	65
1-2 per week	90	26
Several times per week	26	7
Once a day	2	1
Several times a day	5	1
**Consumption of caffeinated beverages**		
No	85	24
1-2 per week	48	14
Several times per week	65	19
Once a day	61	17
Several times a day	92	26
**Consumption of cigarettes/e-cigarettes**		
No	265	75
Yes	86	25
**Consumption of drugs**		
No	327	93
Yes	24	7

**Table 3:** Distribution of Scores for Perceived Workload and PHQ-4 (Depression and Anxiety) of the Sample (n=351)

	n	% of Total
Workload		
0 (low)	43	12
1 (moderate)	133	38
2 (high)	140	40
3 (very high)	35	10
PHQ-4		
0-2	168	48
3-5	112	32
6-8	47	13
9-12	24	7

### Sleep Timing, Sleep Duration, and Sleep Quality

#### Difference in Sleep Timing and Sleep Duration Between Workdays and Work-Free days

**Figure 1:**
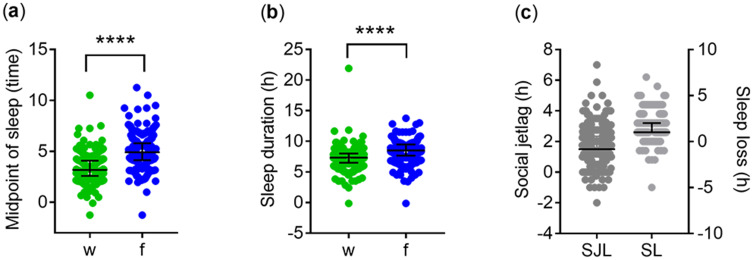
MCTQ-derived variables. (**a**) Midpoint of sleep in local time (hours) on workdays (w) and work-free days (f), (**b**) Sleep duration in hours, (**c**) Social jetlag (SJL) and sleep loss (SL) calculated based on the discrepancy in the midpoint of sleep and sleep duration between w and f, respectively. Middle lines and error bars represent the medians with interquartile range (Q1-Q3). Wilcoxon matched pairs signed rank test, ****p<0.0001, (n=351).

The distribution of MCTQ-derived variables, such as the midpoint of sleep and sleep duration, is shown in Figure 1. The midpoint of sleep (MS) was significantly later (Figure 1a) and sleep duration was significantly longer (Figure 1b) on work-free days than on workdays. This discrepancy in the timing and duration of sleep between workdays and work-free days was reflected in the measures of social jet lag (SJL) and sleep loss (SL), respectively (Figure 1c).

#### Difference in Sleep Quality Between Workdays and Work-Free days

**Figure 2:**
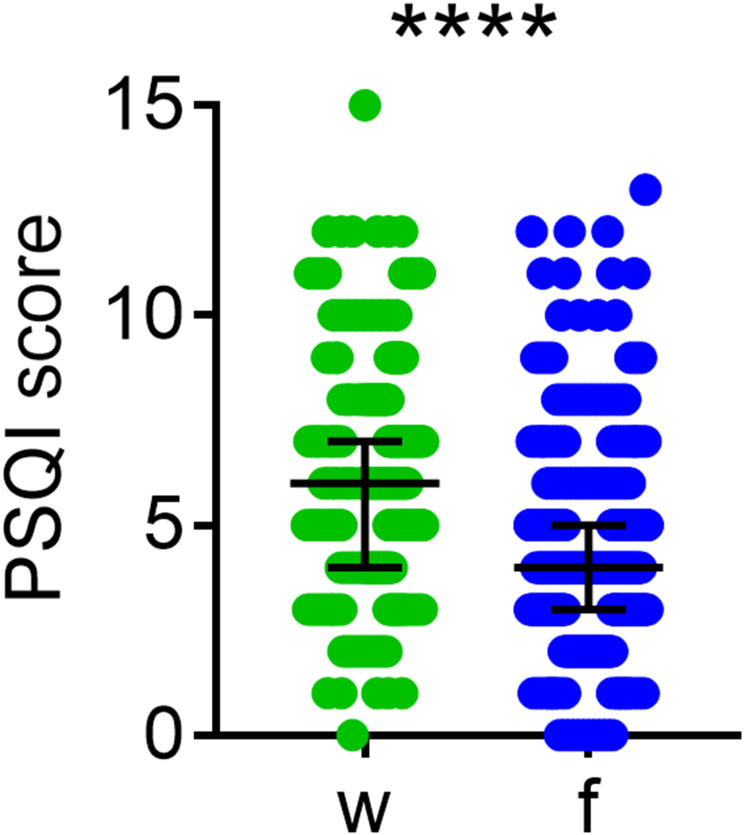
Difference in global PSQI between workdays (w) and work-free days (f) in the sample (n=351). Middle lines and error bars represent the medians with interquartile range. Wilcoxon matched pairs signed rank test, ****p<0.0001, (n=351).

The distribution and differences in the PSQI (global) scores between workdays and work-free days are shown in Figure 2. The PSQI score was significantly lower on work-free days than on workdays, indicating better sleep on work-free days.

The scores for tiredness upon waking (P<0.0001), C1 (subjective sleep quality, P<0.0001), C2 (sleep latency, P<0.0001), C3 (sleep duration, P<0.0001), C6 (use of sleep medication, P=0.026), and C7 (daytime dysfunction, P<0.0001) were significantly lower on work-free days than on workdays, indicating better sleep quality and quantity, as well as lower use of sleep medication and better daytime performance on work-free days. In addition, sleep efficiency (ie, the percentage of time spent asleep while in bed) was significantly higher on work-free days than on workdays (P<0.0001), despite no differences in the C4 score derived from sleep efficiency.

Although the C5 (sleep disturbances) score was not different between workdays and work-free-days (P=0.31), the scores for the C5 subcomponents C5b (wake up in the middle of the night or early morning, P<0.0001), and C5h (have bad dreams, P<0.01) were significantly lower on work-free days, while C5d (cannot breathe comfortably, P<0.03) was significantly higher on work-free days.

#### Associations Among Sleep Quality Variables

**Figure 3:**
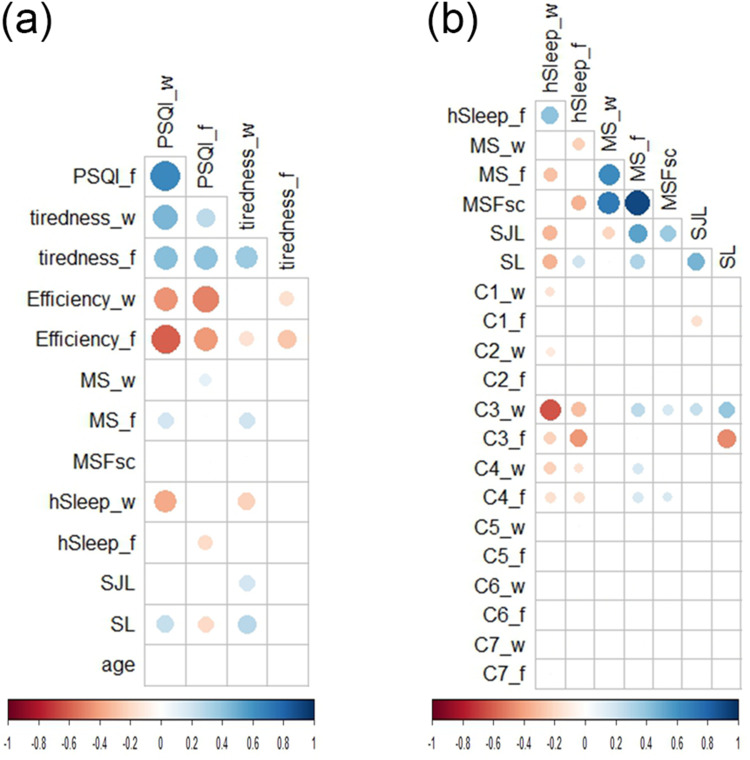
Correlations between sleep quality, timing and duration. Dots in the matrices illustrate Spearman correlation coefficients (*r*), which were significant (P<0.05) after Holm-Bonferroni correction. The size of the dots corresponds to the strength of the correlation. Sleep variables were expressed separately for workdays (_w) and work-free days (_f). (**a**) Correlations between global PSQI and tiredness upon waking with sleep efficiency (percentage of time spent asleep while in bed), other sleep-related variables, and age; (**b**) Correlations between variables related to sleep timing, duration, and PSQI components.

**Abbreviations**: hSleep, sleep duration; MS, midpoint of sleep; MSFsc, midpoint of sleep on work-free days corrected for sleep loss; SJL, social jet lag; SL, sleep loss; tiredness, tiredness upon waking. (n=351).

The PSQI (global) score and tiredness upon waking correlated positively (Figure 3a). PSQI and tiredness upon waking correlated negatively with sleep efficiency (%) (Figure 3a). While tiredness upon waking correlated most strongly with C1 (subjective sleep quality), and C7 (daytime dysfunction) (Supplementary Figure 2a), there was also a positive correlation with various other PSQI components (Supplementary Figure 2a) as well as with C5 (sleep disturbances) subcomponents (Supplementary Figure 2b). Daytime dysfunction also correlated positively with various PSQI components (Supplementary Figure 2c) and C5 subcomponents (Supplementary Figure 2d) in particular C5b (wake up in the middle of the night or early morning) and C5h (bad dreams) on workdays (Supplementary Figure 2d).

#### Associations Among Sleep Quality, Timing and Duration

PSQI and tiredness upon waking on workdays correlated positively with MS on work-free days and SL, and negatively with sleep duration on workdays (Figure 3a). SL correlated positively with PSQI on workdays and negatively with PSQI on work-free days (Figure 3a). As expected, sleep duration was most strongly associated with PSQI components related to sleep quantity (C2, C3, C4) but also with better workday subjective sleep quality (C1) (Figure 3b). MSFsc, SJL, and SL correlated positively with C3 (sleep duration) on workdays (Figure 3b). SJL was also associated with better subjective sleep quality on work-free days (C1) and with tiredness on workdays.

#### Associations Between Anxiety/Depression, Perceived Workload and Sleep Quality

**Figure 4:**
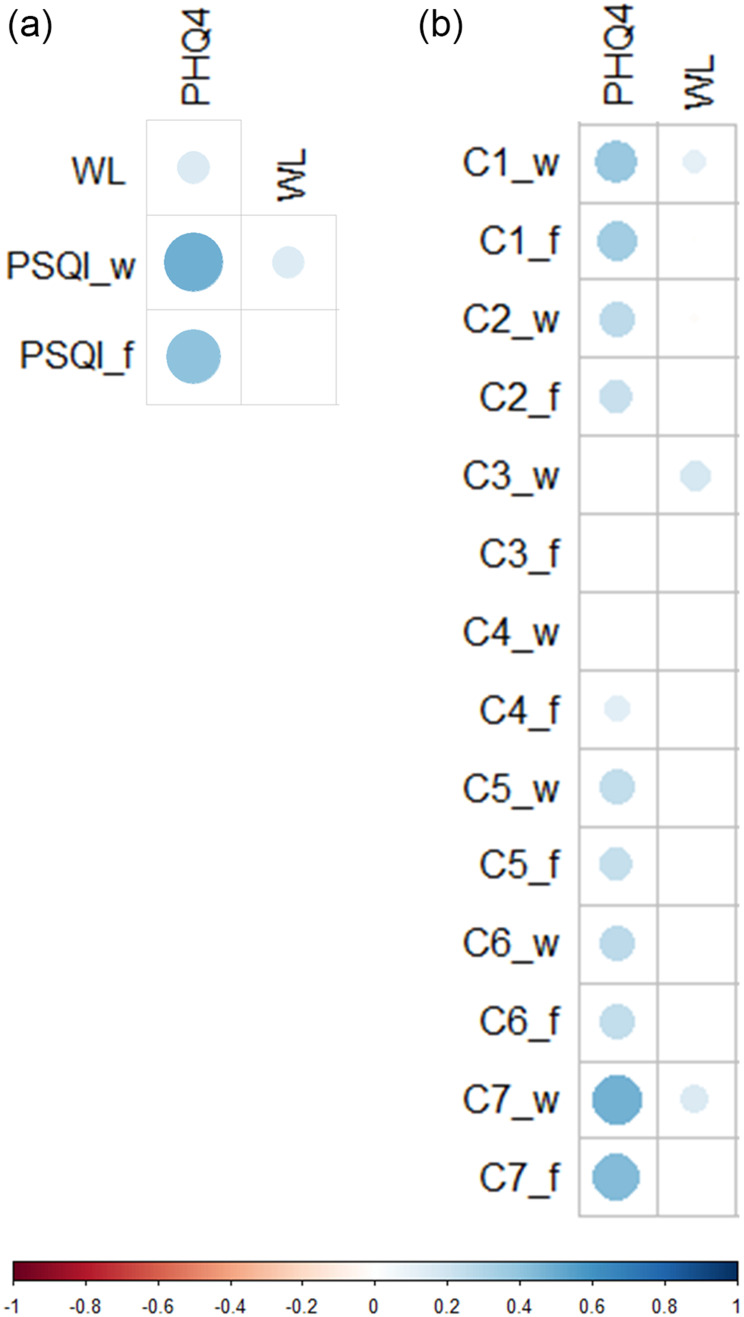
Correlations between anxiety/depression (PHQ-4), perceived workload (WL) and sleep quality (PSQI). Dots in matrix illustrate Spearman correlation coefficients (*r*), which were significant (P<0.05) after Holm-Bonferroni correction. The size of the dots corresponds to the strength of the correlation. PSQI and its components were expressed separately for workdays (_w) and work-free days (_f). Correlations between anxiety/depression (PHQ-4) and perceived workload (WL) with (**a**) each other and the global PSQI, (**b**) PSQI components. (n=351).

The PHQ-4 correlated positively with the global PSQI score and tiredness upon waking, indicating an association between anxiety/depression and poorer sleep quality (Figure 4a). The PHQ-4 correlated positively with various PSQI components including C1 (subjective sleep quality) and C7 (daytime dysfunction) (Figure 4b) as well as with C5 (sleep disturbances) subcomponents (Supplementary Figure 3a). Subjective workload (WL) also correlated positively with global PSQI on workdays (Figure 4a), in particular with C3 (sleep duration) and C7 (Figure 4b). Finally, PHQ-4 scores and WL also correlated positively (Figure 4a).

#### Associations of Lifestyle Factors with Sleep Quality and Anxiety/Depression

**Figure 5:**
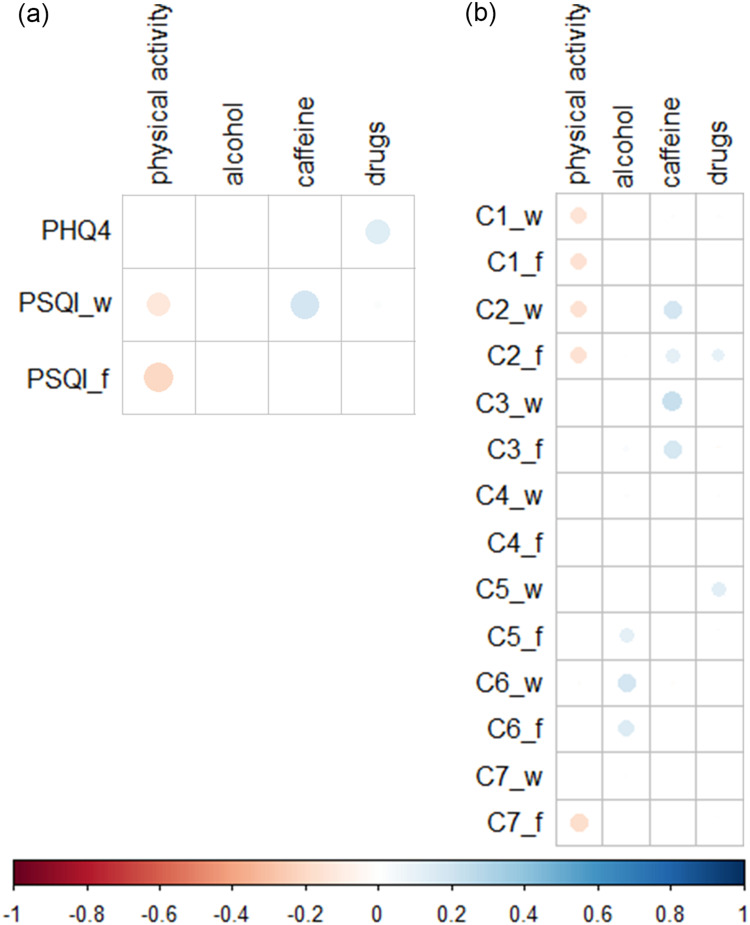
Correlations between lifestyle factors, anxiety/depression (PHQ-4), and sleep quality (PSQI). Dots in matrix illustrate Spearman correlation coefficients (*r*), which were significant (P<0.05) after Holm-Bonferroni correction. The size of the dots corresponds to the strength of the correlation. PSQI and its components were expressed separately for workdays (_w) and work-free days (_f). Correlations between lifestyle factors and (**a**) PHQ-4 and global, (**b**) PSQI components. (n=351).

Physical activity correlated negatively with global PSQI on workdays and work-free days (Figure 5a), in particular with C1 (subjective sleep quality) on work-free days, C2 (sleep latency), and C7 (daytime dysfunction) (Figure 5b).

Consumption of alcoholic beverages or drugs was not significantly correlated with PSQI (Figure 5a), only with some C5 subcomponents (sleep disturbances) (Supplementary Figure 3b). However, consumption of drugs correlated positively with PHQ-4 scores (Figure 5).

Consumption of caffeinated drinks correlated positively with PSQI on workdays (Figure 5), particularly with C2 and C3 (sleep duration) (Figure 5b). Caffeine consumption also correlated negatively with sleep duration, as measured by the MCTQ (Supplementary Figure 1b).

Smoking was associated with higher PSQI on work-free days (, P =0.0002, Wilcoxon test with Bonferroni correction). In particular, with C1 (workdays, P<0.05; work-free days, P=0.03; Wilcoxon test with Bonferroni correction) and C2 (workdays, P=0.004; work-free days, P=0.002; Wilcoxon test with Bonferroni correction).

### Network of Sleep Quality with Anxiety/Depression and Lifestyle Factors

**Figure 6:**
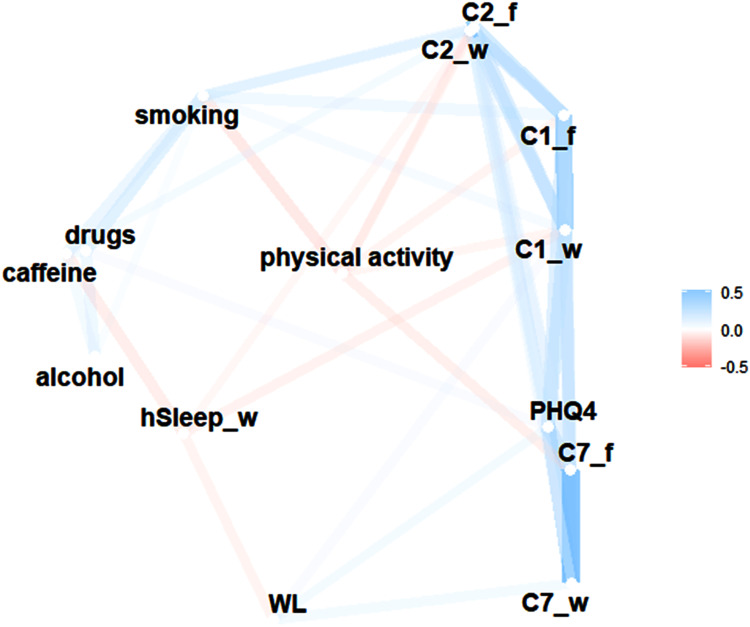
Summery of significant associations between anxiety/depression (PHQ-4), PSQI components, perceived workload (WL) and lifestyle factors. Proximity of the points was determined using multidimensional clustering. Highly correlated variables appear closer together and are joined by stronger paths. The paths are colored according to the direction of the association.

**Abbreviations**: C1, subjective sleep quality; C2, sleep latency; C7, daytime dysfunction. workday sleep duration, _f, work-free days; _w, workdays. (n=351).

The correlation-based network illustrates the associations between anxiety/depression, PSQI components, lifestyle factors, workday sleep duration, and perceived workload (WL) (Figure 6). Anxiety/depression (PHQ-4) was associated with WL, subjective sleep quality (C1), sleep latency (C2) and daytime dysfunction (C7) on both workdays and work-free days. Lifestyle factors such as physical activity and smoking were negatively and positively associated with these PSQI components, respectively. Workday sleep duration was negatively associated with WL, as well as C1 and C2 on workdays. WL was associated with C7 on workdays.

## Discussion

In accordance with previous studies in the general population^21^ and in patients with sleep disorders,^44^ in our cohort of young adults from a large metropolitan area, global PSQI scores were significantly higher on workdays than on work-free days, indicating poorer self-reported sleep quality. In particular, PSQI component scores for subjective sleep quality (C1) and quantity (C2, C3), sleep medication consumption (C6) and daytime dysfunction (C7) were higher on workdays, similar to the study by Pilz et al 2018.^21^ In addition, tiredness upon waking, a parameter used in previous studies as a proxy for sleep quality,^21^ was also higher on workdays. Moreover, sleep efficiency (%), which was negatively associated with global PSQI score, was higher on work-free days.

Interestingly, the scores of the C5 (sleep disturbance) subcomponents “wake up in the middle of the night or early morning” and “have bad dreams” were significantly higher on workdays. Waking up during the night is fairly common^45^ and most often occurs in the early morning, which may be due to decreased homeostatic sleep pressure and endogenous body rhythms. The levels of the waking hormone cortisol,^7^ which is also associated with stress, increase at this time, while those of the sleep hormone melatonin decrease.^17^ Reasons or mechanisms explaining the higher scores we found for “waking up in the middle of the night or early in the morning” on workdays as compared to work-free days remain to be clarified.

In our study, perceived workload was associated not only with self-reported sleep quality on workdays, but also with anxiety/depression and daytime dysfunction on workdays. Previous studies already suggested that stress and workload are associated with sleep quality^13^,^46^ and with nightmares,^47^ which in turn are associated with other daytime effects on well-being, such as anxiety and depressive symptoms.^47^ In line with previous literature, we also found poor sleep quality to be associated with higher scores for anxiety and depressive symptoms. One could speculate that stress reduction strategies might help to break a vicious circle between poor sleep, depressive symptoms and daytime dysfunction.

Chronotype may also contribute to the difference in sleep quality between workdays and workfree days. Later chronotypes have difficulty falling asleep early, which limits the amount of time they can sleep if they start work or study early the following morning. This results in social jet lag and sleep debt, which may be compensated on work-free days.^48^,^49^ Consistent with previous findings, sleep duration was significantly longer on work-free days in our study. SL correlated with higher PSQI scores on workdays and lower PSQI scores on work-free days. SJL also correlated with a lower C1 score. This suggests that on work-free days, longer sleep duration and later mid-sleep were associated with better subjective sleep quality. On workdays, shorter sleep duration was associated with higher tiredness upon waking and PSQI scores, in particular with components related to sleep quantity but also with subjective sleep quality (C1). This suggests that on workdays shorter sleep duration is associated with various aspects of self-reported sleep quality, which is consistent with previous findings suggesting that shorter sleep duration on weekdays may also influence sleep architecture.^21^ Moreover, shorter workday sleep duration was associated with perception of higher workload, consistent with our previous study in medical students.^37^ Due to the cross-sectional design and the subjective nature of workload assessment, direction of causality remains unknown. Sleeping less may increase perceived workload through fatigue or stress, for example, or, conversely, higher workload may lead to shorter sleep due to time constraints. A third factor, like anxiety, may also influence both, or bidirectional influences may coexist. In the latter case, one could speculate that not only reducing actual workload, but also promoting strategies to extend sleep duration, particularly on workdays, could defuse a reinforcing pattern involving poor sleep, perception of high workload, and anxiety/depressive symptoms. Work start times could be adapted to individuals’ predisposition in sleep time/chronotype to ensure sufficient sleep quantity and quality throughout the week. Furthermore, strategies to advance sleep onset could be beneficial, such as reducing exposure to artificial light at night and avoiding high-calorie meals close to bedtime (eg, 50).

In contrast to a study in a larger cohort,^31^ we found no direct correlations between chronotype and consumption of alcohol, nicotine, or caffeine. However, the consumption of these substances was associated with various aspects of self-reported sleep quality, without obvious differences between work and work-free days: Alcohol consumption was associated with the use of sleep medication as well as with various sleep disturbances. This finding is consistent with the modulation of various neurotransmitter systems implicated in insomnia^51^ by alcohol. Consumption of caffeinated drinks was associated with shorter sleep duration and with higher scores in PSQI components on sleep quantity, consistent with caffeine antagonizing the sleep-promoting effects of adenosine.^51^,^52^ Smoking was associated with sleep latency (C2) and subjective sleep quality (C1). This is consistent with a previous study that reported that smokers are twice as likely to experience sleep disorders, with increased sleep latency and daytime sleepiness being the most common issues.^53^ Moreover, smoking appears to decrease slow-wave sleep and increase REM latency.^51^,^53^

While smoking, caffeine and alcohol use were generally associated with poorer subjective sleep quality, physical activity was associated with lower PSQI, in particular with subjective sleep quality (C1), sleep latency (C2), and daytime dysfunction (C7). The positive effects of exercise on sleep, daytime functioning, and quality of life are well documented^54^ and are probably partly explained by an increased release of various neurotransmitters and neurotrophic factors,^55^ stabilization of the circadian system^56^ and modulation of the immune system including microglia.^55^,^57^ Taken together, these results support that a healthy lifestyle,^58^ particularly not smoking and exercising more frequently, can promote sleep quality. Considering the associations between PSQI scores and the PHQ-4, but keeping in mind the caveats related to causality mentioned above, improving sleep quality may also result in lower symptoms of anxiety and depression.

Our study has some limitations. For example, our cohort was relatively small and not representative of the general population, which limits generalizability. As this was a questionnaire-based study, we did not collect any objective or longitudinal data. The use of categorical scoring in the questionnaires simplifies a continuum of behaviors and symptoms. Yet, the use of questionnaires allowed us to gather a large sample with information across different domains (eg, sleep, depressive and anxiety symptoms, lifestyle). Future studies in a large cohort that combine questions about subjective sleep quality with longitudinal sleep data under natural conditions could help develop objective markers of sleep quality. A related limitation is the retrospective nature of the PSQI and the fact that it assesses sleep quality over a period of four weeks, which may introduce, for example, some recall or recency bias. Additionally, work- and work-free days versions of the PSQI could be further validated. Furthermore, as previously discussed, the correlations used here provide an estimation of the strength and direction (positive or negative) of relationships but not causality. Finally, the sample size was calculated to compare PSQI score on work- and work-free days. Therefore, results investigating the associations between sleep quality, its components, lifestyle, as well as depression and anxiety should be considered exploratory, due to limited power and/or increased risk of chance findings, and interpreted accordingly.

## Conclusion

Our data show differences in self-reported sleep timing, duration and quality between workdays and work-free days, corroborating and extending the existing literature. Associations between lifestyle factors (ie, physical activity and substance consumption) and self-reported sleep quality and/or its components across both work- and work-free days suggest that promoting healthier lifestyles could generally improve sleep. Similarly, we found associations between self-reported sleep quality and anxiety/depressive symptoms. While none of our results can establish causality, these findings are consistent with the current body of evidence indicating bidirectional, mutually reinforcing relationships between sleep and mental health. Further associations among self-reported sleep duration, perceived workload and anxiety/depressive symptoms underline the potential of strategies aimed at reducing workload, adjusting work schedules according to chronotype, or advancing sleep duration to improve sleep quality and mental health. Since our study is observational and cross-sectional in nature, further testing of such strategies in experimental intervention studies is warranted.

## Data Availability

Data will be made available on reasonable request from the corresponding author.
